# Supplementary material for: Comparative multiomics analysis of cell physiological state after culture in a basket bioreactor
Source: Sci Rep. 2022 Nov 23;12:20161. doi: 10.1038/s41598-022-24687-4 (PMC9686226; doi:10.1038/s41598-022-24687-4)
Supplement: Supplementary file 1 — Supplementary Information 1. [file 41598_2022_24687_MOESM1_ESM.zip › raw data/Transcriptomic raw data/6.Enrichment/2.KEGG/TE1vsTC1/UP/src/csab05418.html]

csab05418


- csab:103218702

- Up regulated genes

103218702(1.278896182076)

- csab:103222156

- Up regulated genes

103222156(1.83501644039466)

- csab:103243277

- Up regulated genes

103243277(1.88743299439371)

- csab:103243277

- Up regulated genes

103243277(1.88743299439371)

Close
